# Adaptation of the Short Dark Triad (SD3) to Spanish Adolescents

**DOI:** 10.3390/ejihpe14060105

**Published:** 2024-06-04

**Authors:** María Penado Abilleira, María-Luisa Rodicio-García, María-Paula Ríos-de-Deus, Tara Alonso del Hierro

**Affiliations:** 1 Faculty of Health Sciences, International University of La Rioja (UNIR), 26006 Logroño, Spain; taramaria.alonso@unir.net; 2 Grupo de Investigación FORVI (Formación y Orientación para la Vida), Department of Specific Didactics, Research, and Diagnose Methods, Universidade da Coruña, 15071 A Coruña, Spain; m.rodicio@udc.es (M.-L.R.-G.); paula.rios.dedeus@udc.es (M.-P.R.-d.-D.)

**Keywords:** Machiavellianism, narcissism, psychopathy, adolescents, dark triad

## Abstract

(1) Background: The dark triad refers to a personality configuration mainly characterized by the presence of Machiavellianism, narcissism, and psychopathy. Even though adolescence is a critical stage in the development of dark triad traits, to date, this construct has not been studied among adolescents, mainly due to the lack of a measurement instrument adapted to this population. (2) Methods: Using a sample of 1642 adolescents, an adaptation of the Short Dark Triad (SD3) is proposed for this population. To this end, we performed a confirmatory factor analysis of the scale and examined its reliability and the intensity of the dark triad components by sex and sexual orientation. (3) Results: The adapted version of the scale (The Short Dark Triad—Adolescent Version; SD3-A) yielded good psychometric results. Confirmatory factor analysis corroborated the theoretical model of the three factors of dark personality. The results confirmed the greater presence of dark traits in male adolescents, and differences were observed based on sexual orientation. (4) Conclusions: The Short Dark Triad—Adolescent Version (SD3-A) is an effective and comprehensive instrument for the estimation of dark traits in adolescents and can be used as a screening test for this population.

## 1. Introduction

The dark triad refers to a personality configuration mainly characterized by the presence of three specific traits: Machiavellianism, narcissism, and psychopathy [1]. Researchers have justified the grouping of these factors by highlighting that all three traits imply a socially malevolent character with behavioral tendencies toward self-promotion, emotional coldness, duplicity, and aggressiveness (p. 557); thus, they constitute a personality type associated with serious problems or social maladjustments in adults, such as financial fraud [2], bullying or cyberbullying [3,4], and inappropriate behaviors at work [5,6].

To measure the constructs that comprise the dark triad, specific scales from broader instruments have been used for psychopathy [7,8], Machiavellianism [9], and narcissism [10,11]. For evaluation, these scales require completing long questionnaires to acquire a final and precise measurement of the dark triad components. To facilitate the joint measurement of the three constructs, two brief questionnaires that facilitate the simultaneous estimation of these three dimensions have been developed. These questionnaires—the Dirty Dozen [12] and Short Dark Triad [13]—have been widely disseminated and supported at an international level.

The Dirty Dozen is the shorter of the two instruments, comprising only four items for each dimension of the dark triad. Various authors have indicated that this brevity does not fully capture the characteristics of the dark triad traits [14,15,16] despite some reports pointing to the good reliability of this instrument [17,18]. Considering the limitations of the Dirty Dozen, Jones and Paulhus [13] developed the SD3 as a valid and reliable instrument for measuring dark triad traits. This scale consists of 27 statements, 9 for each of the constructs, which are rated on a Likert-type scale with five responses. Jones and Paulhus presented five different studies for validating the scale, including information on the validation of its subscales. 

The analysis involving a comparison of both instruments (DD-SD3) highlighted the greater convergence and incremental validity of the SD3 in terms of longer and more established measures of the dark triad traits, adequate discriminant validity, and criterion validity concerning the traits of the five-factor model [19]. Due to these superior psychometric properties, the SD3 has been widely propagated worldwide [3,20,21], including adaptations and their analyses in countries as diverse as Germany [22], Argentina [23], China [24], Poland [25], Croatia [26], Russia [27], Iran [28], Japan [29], Taiwan [30], Holland [31], Serbia [32], Thailand [33], France [34], Turkey [35], and Spain [36].

Although several studies have involved psychometric analyses of the SD3 in different adult populations, studies on dark triad traits in the adolescent population are scarce, primarily due to the lack of adaptation and validation of this instrument for this age group. Studies on the adaptation of the SD3 in the Italian adolescent population indicate good reliability of the original unmodified scale [37], while disparate results have been obtained regarding Russian adolescents [38], with items that can be associated with more than one factor (especially in the psychopathy dimension). To date, the only attempts to adapt the original scale for minors were conducted in an excessively small sample of Polish adolescents [39] and a sample of Portuguese adolescents in disadvantaged and impoverished zones in the Lisbon area [40]. 

In the study carried out among Polish adolescents, the scale was refined through several adjustments considering various criteria, namely through the arbitrary elimination of one of its items (“I enjoy having sex with people I hardly know”), internal validation based on statistical criteria, and external validation based on the correlation found between other instruments that do not estimate the presence of dark traits such as the Big Five Personality Test, the Buss–Perry Aggression Questionnaire, or the Rosenberg Self-Esteem Scale. The final solution proposed by the authors of 25 items has not been validated for the adolescent population, nor are there any reliability or descriptive results regarding its application to the adolescent population. For the Portuguese adaptation of the Short Dark Triad, researchers started from all the items on the scale based on exclusively statistical criteria (low loadings and low corrected item–total correlations) to eliminate two items from each one of the factors. The results revealed good reliability and a high correlation between the final scale and similar instruments (such as the Dirty Dozen). As indicated in a previous study [40], this was the first attempt to validate the SD3 for use among Portuguese-speaking youth and one of the few studies examining dark triad traits among youth under 18 years old.

Although these two previous studies have been the only attempts thus far regarding the adaption of the Short Dark Triad to the adolescent population, they have important limitations that must be considered to generalize their results and the proposed instruments. In the study carried out among Polish adolescents, the item selection process was compromised from the beginning as the study did not start from the complete original scale used in the same way. Additionally, although statistical analyses were carried out to study the structure of the instrument, concurrent validation was performed using general and non-specific dark personality instruments, thus preventing the corroboration of the proposed scale with the measures obtained by other instruments. Regarding the research focused on Portuguese adolescents, the sample was not representative of the adolescent population in general, as its 412 participants were selected from public schools in disadvantaged areas of the Lisbon area, which have distinct characteristics (low status socioeconomic, unfinished education, problems with the law, etc.) that are not representative of the adolescent population. Finally, it should be noted that the authors use non-scaled instruments for the concurrent validation of the proposed scale for the adolescent population, thus limiting the conclusions of the study.

Taken together, these limitations highlight the need to corroborate whether the differences in dark triad traits in the adult population begin during adolescence, which will facilitate a better understanding of the emergence of the phenomenon or how it is expressed in its initial stages. In terms of sex differences, studies indicate that males receive higher scores than females in the analysis of the three dark triad traits in both adults [18,41,42] and adolescents [40,43]. Regarding differences in sexual orientation, to date, no investigations have been conducted on adolescents; however, the results for the adult population indicate that bisexual women scored higher than heterosexual women or lesbians on the three traits [44,45], while homosexual men scored lower than heterosexual men [46].

Considering the previous evidence, it is necessary to develop an assessment instrument for analyzing dark triad traits in the adolescent population that enables a rapid estimation of the magnitudes of these constructs. The present study aimed to examine the psychometric properties and factorial structure of the SD3 among Spanish adolescents and propose an adaptation that considers the characteristics of this population and simultaneously allows for the valid and effective measurement of the dark triad traits in minors.

## 2. Materials and Methods

### 2.1. Participants

In total, 1642 adolescents who were enrolled in centers that provide Compulsory Secondary Education (ESO) and Baccalaureates in Galicia (Spain) participated in this study, comprising 48.2% (792) boys, 51.5% (845) girls, and only 0.3% (5 people) not identifying their sex. The age range was between 11 and 19 years (M = 15.08; SD = 1.62), with the mean age for girls (15.18) higher than that for boys (14.97) (t (1642) = −2.612; *p* < 0. 01). The analysis of the age group distribution revealed a homogeneous sample, with the largest group being adolescents aged 15 and 16 but without major differences among the age groups based on sex (Table 1).

In addition to sex and age data, the participants were asked to indicate their sexual orientation. The responses revealed that half of the adolescents identified as exclusively heterosexual (51.6%), 35.2 % were exclusively homosexual, 10% were bisexual, and 3.2% identified as other. These results differ from the latest national statistics published by the Youth Institute of the Ministry of Youth and Children of the Government of Spain in 2020 [47], which indicated that 16% of youth between 15 and 29 years old identify as homosexual or bisexual, 77.5% as heterosexual, less than 0.5% without attraction to any sex, and 5.6% do not know or do not answer. Nevertheless, this population involves complicated and disparate age groups. They all fit into the category of adolescents, but an 11-year-old is not the same as a 19-year-old. This was also highlighted when investigating, for example, the differences between the 11–13-year-olds and other age groups. It was observed that younger children consider themselves to be heterosexual more than those aged 14–19 years old.

A more in-depth analysis of the results of sexual orientation based on sex reveals significant differences (X^2^(2) = 680.188; *p* < 0.01), indicating that adolescent girls more frequently report feeling attracted to people of both sexes than boys of the same age, who have more tendency to identify as heterosexual (Table 2). These results are consistent with national statistics.

### 2.2. Instruments

The SD3 [13] has been specifically designed to measure the three dark triad dimensions (Machiavellianism, narcissism, and psychopathy). The scale comprises 27 items (9 for each dimension), for which the participants must indicate their degree of agreement with five response options rated on a Likert-type scale, ranging from “totally disagree” to “totally agree.” These items include statements that are indicative of psychopathic (people who mess with me always regret it), narcissistic (people see me as a natural leader), and Machiavellian (it is not wise to tell your secrets) traits. Of the 27 items, 5 statements were worded in the opposite direction (items 11, 15, 17, 20, and 25) and had to be recoded to obtain the final score. The higher the scores obtained on the scale, the higher the levels of dark triad traits.

### 2.3. Procedure

For data collection, the scale was digitized using Google Forms (https://www.google.com/forms, accessed on 15 February 2024). Before collecting participants’ data, informed consent was obtained from all parents after providing information on the objectives of the intervention, the instruments used, and the sociodemographic data required. For obtaining consent from minors, information on research objectives, the research team, and the data protection compliance clause were included on the first page of the questionnaire. In addition to the informed consent, the participants were required to agree with the research objectives and response confidentiality. No personal data leading to participants’ identification were collected, and the study design was approved by the ethics committee of our university. This study complied with the recommendations of the Declaration of Helsinki. 

The researchers were present in the classrooms during the application of the scale, fully explaining the items and ensuring that no one responded without understanding the question being asked. The scale was translated by a professional English philologist with in-depth knowledge of Spanish, having been in this country since the age of 15. Once translated, one adolescent from each age group was asked to complete the scale, which helped to relatively modify the translation, adopting expressions that were more comprehensible to them without changing the meaning of the text.

### 2.4. Data Analysis

Data analysis was performed by verifying the normal distribution of the sample using the Kolmogorov–Smirnov statistic. Once it was verified that the sample did not follow a normal distribution, the internal consistency of the instrument was evaluated using Cronbach’s alpha coefficients. To evaluate the internal structure of the scale, confirmatory factor analysis (CFA) was conducted using the AMOS software (v.23. IBM Corp., Chicago, IL, USA). For estimation, the unweighted least squares (unweighted least squares; ULS) method was utilized due to the ordinal nature and non-normal distribution of the studied variables [48,49]. The following indices were used to assess the fit of the model: goodness-of-fit index (GFI), adjusted goodness-of-fit index (AGFI), root-mean-squared residual (RMR) index, root-mean-square error of approximation (RMSEA), normed fit index (NFI), and relative fit index (RFI). According to Kline [50], values indicate a good fit if RMR < 0.06 and RMSEA < 0.05 and GFI, AGFI, NFI, and RFI > 0.90. Following the most current recommendations [51], the Comparative Fit Index (CFI) and Tucker–Lewis Index (TLI) have been obtained to analyze the robustness of the model. According to recent investigations [52,53], authors indicate a score greater than 0.90 to consider a good fit for the model and scores greater than 0.95 are considered to have an optimal fit.

In addition, structural education models (ESEMs) were used to assess the consistency of the final scale, as they are a flexible and useful alternative for this type of study and can be compared to the CFA results. No differences were observed, confirming the consistency of the analysis and facilitating the selection of the items included in the final scale since, in both cases, the same fit is maintained. This allowed us to rule out any poorer-fit item due to the statistical procedure used.

Finally, for comparisons between the subscales, a mean difference analysis (Pearson’s χ2) with post hoc tests was performed using the Kruskal–Wallis statistic. The effect size of the differences was estimated following the recommendations of Cohen [54] (1988), which reports the following intervals for r: 0.1 to 0.3: small effects; 0.3 to 0.5: intermediate effects; 0.5 and higher: strong effect. The descriptive data and internal consistency were determined through CFA using IBM SPSS Statistics (v.25, IBM Corp., Chicago, IL, USA) and AMOS extension (v.23) software.

## 3. Results

### 3.1. Reliability

The reliability analysis of the Short Dark Triad (SD3) among Spanish adolescents yielded a Cronbach’s alpha coefficient of 0.828, which increased if some statements were eliminated, especially those that were worded in the opposite direction. Improvements were observed regarding both the psychometric results of the total scale and the reliability of the subscales of Machiavellianism (α = 0.705), narcissism (α = 0.609), and psychopathy (α = 0.701).

After eliminating the statements that limited the instrument’s reliability, a final scale consisting of 18 elements was achieved; in this scale, each of the dark triad constructs comprised six statements rated on a Likert-type scale with five responses. The adaptation of the scale for adolescents (The Short Dark Triad—Adolescent Version (SD3-A) (Appendix A) did not include any statements worded in the opposite direction that would require modification or recoding.

The reliability analysis of the adapted scale (The Short Dark Triad—Adolescent Version (SD3-A) showed a Cronbach’s alpha coefficient of 0.854, which improved the results obtained globally on the unmodified scale and demonstrated good psychometric results regarding the Machiavellianism (α = 0.691), narcissism (α = 0.645), and psychopathy (α = 0.768) subscales of the instrument. Finally, the statistical results of the adapted scale (The Short Dark Triad—Adolescent Version (SD3-A) confirmed that eliminating some of the statements, in addition to those already established, did not lead to an improvement in the reliability of the scale or the fit of the model (Table 3).

### 3.2. Factorial Structure

As with the reliability analyses, the factorial structure of the original scale was assessed to determine whether the unaltered scale presents adequate goodness-of-fit indices or whether the scale requires restructuring to adapt it to the adolescent population. An initial analysis of the unmodified scale yielded inadequate fit indices according to the established standards, with a root-mean-squared residual (RMR) index of 0.058 and goodness of fit index (GFI), adjusted goodness of fit index (AGFI), normed fit index (NFI), and relative fit index (RFI) values below 0.90. The factorial analyses of the SD3-A comprising 18 statements revealed a good fit with positive adjustment values for the solution of three factors. Therefore, we concluded that the scale was adequately adjusted for use in the adolescent population (Table 4).

### 3.3. The Presence of Dark Triad Traits 

In addition to the psychometric analyses conducted to validate the SD3 for the adolescent population, the mean scores were obtained for each of the scale’s factors to estimate the intensity of the dark triad traits in minors. In general, Spanish adolescents scored higher in Machiavellianism than in narcissism and psychopathy, with an increase in this factor observed in both boys and girls. Regarding differences by sex, boys scored higher than girls in all factors of the scale, with significant differences and effect sizes for Machiavellianism (*X*^2^(2) = 943,307; *p* <0.01; *r* = 0.32) and psychopathy (*X*^2^(2) = 1253,694; *p* < 0.01; *r* = 0.53) and non-significant differences in narcissism (Table 5).

A general analysis of the participants’ sexual orientation indicated that adolescents who identified as exclusively homosexual received higher scores in the three factors than adolescents who identified as exclusively heterosexual or bisexual. Although homosexual adolescents scored higher than their heterosexual and bisexual counterparts in all three factors, the results of the post hoc tests only indicated significant results for psychopathy (*X*^2^(2) = 14,500; *p* < 0.01; *r* = 0.84), where homosexual adolescents scored higher than exclusively heterosexual adolescents (Table 6).

Among male adolescents, those identifying as exclusively homosexual had a higher score than their exclusively heterosexual or bisexual counterparts. Although this difference was observed in all the traits, the post hoc test results indicated a statistically significant difference in Machiavellianism (*X*^2^(2) = 326,609; *p* < 0.01; *r* = 0.34) and psychopathy (*X*^2^(2) = 521,578; *p* < 0.01; *r* = 0.32) scores between homosexual and exclusively heterosexual boys (Table 7).

Considering female adolescents, a greater presence of Machiavellian (*X*^2^(2) = 458,323; *p* < 0.01; *r* = 0.50) and psychopathic (*X*^2^(2) = 669,670; *p* < 0.01; *r* = 0.51) traits was observed among those who identified as bisexual than among exclusively homosexual or heterosexual females, which was statistically significant. Regarding narcissistic traits, the highest scores were observed in girls of exclusively homosexual orientation, with no significant differences observed in the rest of the groups (Table 8).

## 4. Discussion and Conclusions

The reliability analyses of the SD3-A revealed that its adaptation for the Spanish adolescent population provides better results than those obtained using the unmodified scale in Italian adolescents [37] as well as the version adapted for Polish adolescents [39]. Furthermore, the proposed scale yielded Cronbach’s Alpha scores—both in the global scale and in the narcissism, psychopathy, and Machiavellianism subscales—that were comparable to those obtained by Jones and Paulhus [13] regarding the psychometric properties of the SD3 in adults.

The final adaptation of the scale comprising 18 items (SD3-A) is the shortest of all adaptations for the adolescent population thus far [37,39]. Moreover, the scale analysis confirmed the trifactorial model of the dark triad personality, which has already been obtained through the application of the SD3 in Spanish adults [36], Italian adolescents [37], and Portuguese adolescents in disadvantaged and impoverished zones in the Lisbon area [40]. These results indicate that the adapted version of the SD3 proposed in this study, the SD3-A, is a valid instrument for estimating dark triad traits in adolescents. Adolescence is a critical stage for the development of personality and dark triad traits [43]. Furthermore, the association between dark triad traits and behavioral problems in adolescence and adulthood necessitates a rapid and effective instrument for the detection of these traits in adolescents that would facilitate the design and implementation of specific prevention programs. The association of dark triad traits with problematic behaviors such as high-risk sexual behaviors, including coercion [55], more positive attitudes toward rape [56], repeated sexual advances [57], a greater propensity to commit romantic revenge [58], and greater enjoyment of tormenting others online [59], as well as both the perpetration and victimization of bullying [60], justifies the usefulness of similar instruments that can be easily used by all types of professionals.

The descriptive results indicated average values for dark personality traits among the participants, similar to the results of recent studies involving the adolescent population and young adults [61,62]. Regarding sex differences, male adolescents had higher levels of dark triad traits than female adolescents, confirming the differences based on gender that have been observed cross-culturally in the adult population [56,63,64]. Regarding sexual orientation, significant differences were observed in Machiavellian and psychopathy, for which adolescents with an exclusively homosexual orientation presented higher scores than their exclusively heterosexual counterparts. Although the differences in narcissism scores based on sexual orientation were not statistically significant, higher scores were observed in exclusively homosexual or bisexual adolescents, which may reflect the beginning of differences in the dark triad traits based on sexual orientation that are observed later in adulthood [65].

As observed for the adult population [65], exclusively homosexual male adolescents scored higher in Machiavellianism than their heterosexual and bisexual counterparts, although the differences were not significant, similar to narcissism scores. In contrast to the adult population, a statistically significant increase in psychopathic traits was observed among homosexual male adolescents compared to the rest of the analyzed groups [65]. The scores obtained based on sexual orientation in female adolescents are more consistent with the results obtained for the adult population to date; according to these results, there is an increase in psychopathic and Machiavellian traits among bisexual women in comparison with exclusively heterosexual women [41]. The results of this study, which indicate a greater intensity of dark triad traits in exclusively homosexual male adolescents, seem to contradict, at least in this age group, the gender shift hypothesis, indicating that non-heterosexual men have similarities with heterosexual women [66]. For the adult population, these results can be explained by experiences of discrimination already present during adolescence that manifest as harsher treatment during adulthood [67,68], thus activating an adaptive response involving dark triad traits (manipulation, deception, etc.).

Finally, the results observed in female bisexual adolescents, indicating a greater presence of dark triad traits than homosexual and heterosexual adolescents, are consistent with the results in the adult population [69]. This indicates that the masculinization of behaviors does not occur linearly from heterosexual to homosexual to bisexual women, further demonstrating that some of the more typically masculine traits are more common in bisexual women than in homosexual women [70]. These results can be considered as a starting point regarding the behavior of the dark triad traits in the adolescent population depending on sex and sexual orientation, requiring further research into whether these differences are motivated by other variables apart from the studied and that have already been considered for the adult population, such as gender roles [71].

It has been established that younger individuals are less aware of their sexual tendencies and respond more with what is expected of them or because they are afraid of their intimacy being known rather than with the truth. This should be corroborated in further studies, as it can considered a limitation of this study. Another limitation concerns the methodology used. It is necessary to collect qualitative information for the triangulation of the data and greater validity of the results. In-depth interviews may be appropriate given the intimate nature of the data and would help to better understand the reasons for the results presented here.

In this way, possible biases regarding the age of the subjects or their sexual tendencies could be eliminated. However, let us not lose sight of the fact that this is not the aim of this article. As mentioned in the Introduction, we sought to validate a scale in an adolescent population, given the lack of preliminary studies on this subject; therefore, we could not analyze convergent validity. Despite the good psychometric results captured, it is necessary to point out that the results obtained may be due to other factors apart from the dark triad traits, making it necessary that future research verify the scale measure invariance to rule out idiosyncratic use and interpretive bias. The scale adapted to Spanish adolescents (SD3-A) should be applied in subsequent studies using different samples or populations of adolescents to confirm these results. Thus, the proposed tool presented here will gain greater consistency, both internally and externally.

## Figures and Tables

**Table 1:** Sample distribution by age range.

	Male	Female
	N (%)	N (%)
Less than 12 years	68 (8.4%)	62 (7.1%)
12 to 14 years	234 (28.9%)	205 (23.6%)
15–16 years	343 (42.4%)	384 (44.2%)
17–18 years	155 (19.2%)	216 (24.9%)
More than 18 years	9 (1.1%)	2 (0.2%)

**Table 2:** Sexual orientation results based on adolescents’ sex.

	Male	Female
	N (%)	N (%)
Homosexual	190 (37%)	203 (34.3%)
Heterosexual	296 (57.7%)	281 (47.5%)
Bisexual	18 (3.5%)	92 (15.6%)
Other	9 (1.8%)	15 (2.5%)

**Table 3:** Psychometric properties of the Short Dark Triad—Adolescent Version (SD3-A).

	M-i	SD-i	r^c^_i_-t	α-i
Machiavellianism				
1	40.62	136.330	0.462	0.847
2	40.14	131.374	0.547	0.843
3	40.33	128.775	0.588	0.841
4	39.60	134.170	0.448	0.848
5	40.03	137.157	0.371	0.851
6	39.58	137.468	0.332	0.853
Narcissism				
1	40.67	136.961	0.438	0.848
2	40.56	137.146	0.426	0.849
3	40.68	136.671	0.461	0.847
4	39.45	136.117	0.388	0.851
5	40.46	137.363	0.358	0.852
6	39.30	139.226	0.297	0.853
Psychopathy				
1	40.81	133.374	0.570	0.843
2	40.78	133.394	0.558	0.843
3	40.80	137.584	0.419	0.849
4	40.32	133.358	0.506	0.845
5	40.40	132.836	0.551	0.843
6	40.62	133.197	0.552	0.843

M-i, the mean if the item is removed; SD-i, standard deviation if the item is removed; r^c^_i_-t, total item correlation if the item is removed; α-i, Cronbach’s alpha if the item is removed.

**Table 4:** Psychometric properties of the Short Dark Triad—Adolescent Version (SD3-A).

Index	Results
RMSEA	0.048
RMR	0.058
GFI	0.985
AGFI	0.981
NFI	0.973
RFI	0.968
CFI	0.950
TLI	0.961

RMSEA: Root-Mean-Square Error of Approximation; RMR: Root-mean-squared residual index; GFI: goodness-of-fit index; AGFI: adjusted goodness-of-fit index; NFI: normed fit index; RFI: relative fit index; CFI: Comparative Fit Index and TLI: Tucker–Lewis Index.

**Table 5:** Results obtained regarding dark trait differences according to sex.

		Machiavellianism	Narcissism	Psychopathy
	N	M (SD)	M (SD)	M (SD)
Boys	792	2.69 (0.84)	2.51 (0.74)	2.09 (0.80)
Girls	845	2.48 (0.80)	2.40 (0.73)	1.93 (0.80)
Total	1637	2.60 (0.84)	2.47 (0.75)	2.02 (0.83)

**Table 6:** Results of dark trait analysis according to sexual orientation.

		Machiavellianism	Narcissism	Psychopathy
	N	M (SD)	M (SD)	M (SD)
Homosexual	577	2.86 (0.95)	2.87 (0.92)	2.59 (0.91)
Heterosexual	847	2.46 (0.81)	2.38 (0.75)	1.88 (0.79)
Bisexual	164	2.54 (0.77)	2.40 (0.69)	2.03 (0.83)
Total	1640	2.60 (0.84)	2.47 (0.75)	2.02 (0.83)

**Table 7:** Results of dark trait according to sexual orientation in boys.

		Machiavellianism	Narcissism	Psychopathy
	N	M (SD)	M (SD)	M (SD)
Homosexual	278	3.26 (0.85)	2.93 (0.98)	2.96 (0.82)
Heterosexual	408	2.58 (0.80)	2.49 (0.75)	2 (0.78)
Bisexual	79	2.40 (0.81)	2.29 (0.62)	1.90 (0.71)

**Table 8:** Results of the dark trait among female adolescents according to sexual orientation.

		Machiavellianism	Narcissism	Psychopathy
	N	M (SD)	M (SD)	M (SD)
Homosexual	350	2.33 (0.94)	2.88 (0.95)	1.95 (0.66)
Heterosexual	420	2.31 (0.77)	2.24 (0.71)	1.74 (0.76)
Bisexual	75	2.54 (0.77)	2.41 (0.71)	2.04 (0.86)

## Data Availability

Data are available upon request from researchers who meet the eligibility criteria. Kindly contact the first author privately through e-mail.

## Appendix A

Below are a series of statements that refer to your way of thinking and/or acting.

Please indicate to what extent you agree with each of the following statements based on the following scale:

1. Strongly disagree
2. Disagree
3. Neither disagree nor agree
4. Agree
5. Totally agree

Remember that the questionnaire is ANONYMOUS and no one will have access to it.

Machiavellianism

1. I like to use clever manipulation to get my way.
2. It’s wise to keep track of information that you can use against people later.
3. You should wait for the right time to get back at people.
4. There are things you should hide from other people to preserve your reputation.
5. Make sure your plans benefit yourself, not others.
6. Most people can be manipulated.

Narcissism

1. People see me as a natural leader.
2. Many group activities tend to be dull without me.
3. I know that I am special because everyone keeps telling me so.
4. I like to get acquainted with important people.
5. I have been compared to famous people.
6. I insist on getting the respect I deserve.

Psychopathy

1. I like to get revenge on authorities.
2. Payback needs to be quick and nasty.
3. People often say I’m out of control.
4. It’s true that I can be mean to others.
5. People who mess with me always regret it.
6. I’ll say anything to get what I want.
